# Supplementary material for: Antibody Response to the SARS-CoV-2 Vaccine and COVID-19 Vulnerability during the Omicron Pandemic in Patients with CLL: Two-Year Follow-Up of a Multicenter Study
Source: Cancers (Basel). 2023 May 30;15(11):2993. doi: 10.3390/cancers15112993 (PMC10251854; doi:10.3390/cancers15112993)
Supplement: Supplementary file 1 [file cancers-15-02993-s001.zip › cancers-2372274-supplementary.pdf]

Antibody response to the SARS-CoV-2 vaccine and COVID-19 vulnerability during the Omicron pandemic in patients with CLL: two-year follow-up of a multicenter study.

SUPPLEMENTARY INFORMATION

1. Assessment of the serologic response to the SARS-CoV-2 vaccine.
2. Supplementary Figure S1. Patient disposition.
3. Supplementary Table S1. Factors with an impact on serologic response.
4. Supplementary Table S2. Factors with an impact on the development of COVID-19.

Assessment of the serologic response to the SARS-CoV-2 vaccine.

A centralized assessment of the antibody response was made at the Istituto Superiore di Sanità (I.S.S.) of Rome. Blood samples were taken before the first dose of the vaccine and three weeks after the second and third doses. Additional samples were taken six months from the second dose of the vaccine to evaluate the persistence of IgG antibodies to the SARS-CoV-2 virus.

Methods to assess COVID-19 IgG levels and statistical methods are reported in the Supplementary material.

COVID-19 IgG levels were evaluated using the COVID-19 kit SeroIndex, Kantaro Quantitative SARS-CoV-2 IgG Antibody R.U.O. (R&D System). The system consisted of two serial direct Enzyme-Linked Immunosorbent Assays (ELISA) intended to detect human IgG antibodies to the SARS-CoV-2 virus quantitatively. An initial ELISA assay was performed to test for antibodies reactive to the recombinant Receptor Binding Domain (RBD) of the SARS-CoV-2 Spike protein. Positive specimens were tested using a quantitative ELISA against the full-length SARS-CoV-2 Spike protein. The calibration curve ranged from 3.2 to 160 AU/mL SARS-CoV-2 IgG titers ≥3.2 AU/mL identified patients with serologic response.

Supplementary Figure S1. Patient disposition

Received the SARS-CoV-2 vaccine: 200 patients

- Not eligible for analysis of SR[1]: 4 patients (anti-SARS-CoV-2 IgG antibodies present at baseline, 3; did not receive the second dose of vaccine, 1).
- Lost to the follow-up: 1 patient.

.

Tested for the SR[1] after the second dose of the vaccine: 195 patients

Tested for the SR[1] before the third dose of the vaccine: 76 patients

- COVID-19 after the second dose of the vaccine: 4 patients
- Non-COVID-19 related deaths: 5 patients (infection,1; cardiovascular, 1; Richter transformation, 1: CLL, 1, myeloma, 1).
- Lost to the follow-up: 3 patients.

In follow-up after the second dose of the vaccine:183 patients

Received the third dose of the SARS-CoV-2 vaccine: 179 patients

Tested for the SR [1] after the third dose of the vaccine: 165 patients

Received more than three doses of the SARS-CoV-2 vaccine: 46 patients (four doses, 32 patients, five doses 14 patients)

- COVID-19 after ≥3 doses of the vaccine: 76 patients
  - (after three doses, 61, four doses, 12 patients; five doses, 3)
- COVID-19-related deaths: 3 patients.
- Non-COVID-19 related deaths: 2 patients (Richter transformation, 1; second malignancy, 1).

Alive and COVID-free: 109 patients

- COVID-19: 80 (41%)patients
- COVID-19-non related deaths: 6 (6/200,3%)
- COVID-19-related deaths: 3 (3/200, 1.5%)
- Lost to the follow-up: 4

[1] SR: serologic response

Supplementary Table S1. Factors with an impact on serologic response.

	All patients (N=195)						Patients treated with targeted agents (N=143)					
	Univariable analysis			Multivariable analysis			Univariable analysis			Multivariable analysis		
	OR	95% CI	P value	OR	95% CI	P value	OR	95% CI	P value	OR	95% CI	P value
Gender<br>male *vs.* female	0.92	0.52-1.65	0.80				1.52	0.72-3.24	0.27			
Age, years	**0.94**	**0.92-0.97**	**<0.0001**	**0.93**	**0.90-0.96**	**<0.0001**	**0.96**	**0.92-0.99**	**0.016**	**0.96**	**0.92-0.99**	**0.038**
CIRS<br>≥6 *vs.* <6	0.40	0.21-0.76	0.005				0.74	0.35-1.53	0.41			
Months from CLL to the SARS-CoV-2 vaccine,<br>≥92 *vs.* <92	0.62	0.34-1.10	0.10				0.81	0.39-1.68	0.57			
Lymphocyte count x 10^9/L<br>≥ 5.00 *vs.* <5.00 x10^9/L	1.53	0.86-2.75	0.15				0.84	0.37-1.89	0.67			
IgG levels, mg/dl<br><550 *vs.* >550	**0.27**	**0.14-0.54**	**<0.0001**	**0.28**	**0.13-0.58**	**0.001**	**0.28**	**0.12-0.66**	**0.004**	**0.31**	**0.12-0.79**	**0.014**
Beta-2 microglobulin mg/dl<br>≥ 3.5 *vs.* <3.5	0.41	0.17-0.97	0.04				0.36	0.12-1.15	0.084			
Rai stage<br>III-IV *vs.* 0-II	0.40	0.11-1.50	0.17				0.29	0.04-2.43	0.26			
Clinical signs of progressive disease<br>present *vs.* absent	0.52	0.22-1.23	0.14				*	*	*			
IGHV<br>unmutated *vs.* mutated	0.54	0.27-1.11	0.09				0.65	0.28-1.50	0.32			
*TP*53 deletion and/ or mutation<br>absent *vs.* present	1.28	0.69-2.39	0.43				0.68	0.32-1.48	0.34			
Prior treatment<br>yes *vs.* no	0.22	0.10-0.51	**<0.0001**				NA	NA	NA			
Number of prior treatments<br>≥2 *vs.* 1	0.36	0.19-0.68	**0.002**				0.68	0.33-1.41	0.30			
Months from last rituximab<br>≤12 *vs* no rituximab or >12	**0.12**	**0.04-0.42**	**0.001**	**0.10**	**0.03-0.37**	**0.001**	**0.19**	**0.05-0.66**	**0.009**			
Ibrutinib based-therapy<br>*vs* venetoclax-based therapy							0.78	0.38-1.62	0.51			
Months from the start of Ibrutinib or<br>venetoclax-based therapy ≥ 18 *vs.* <18							**0.18**	**0.08-0.42**	**<0.0001**	**0.17**	**0.06-0.44**	**<0.0001**

Abbreviations: Covid-19, Coronavirus ***disease*** 2019; SARS-CoV-2, severe acute respiratory syndrome *coronavirus 2;* Ig, immunoglobulins; *TP*53 gene, tumor protein p53 gene; Del., deletion; IGHV, immunoglobulin heavy chain variable region gene. * Not evaluable due to the absence of a serologic response in all patients with a progressive disease. NA, not applicable.

Supplementary Table S2. Factors with an impact on the development of COVID-19.

	All patients (N=195)						Patients treated with targeted agents (N=143)					
	Univariable analysis			Multivariable analysis			Univariable analysis			Multivariable analysis		
	HR	95% CI	P value	HR	95% CI	P value	HR	95% CI	P value	HR	95% CI	P value
Gender<br>male *vs.* female	1.21	0.78-1.90	0.40				1.04	0.61-1.75	0.90			
Age, years	**0.97**	**0.95-0.9949**	**0.015**	**0.97**	**0.95-0.9995**	**0.046**	**0.97**	**0.95-0.9974**	**0.030**	**0.97**	**0.94-0.9958**	**0.023**
CIRS<br>≥6 *vs.* <6	1.05	0.66-1.67	0.82				0.99	0.60-1.65	0.99			
Months from CLL to the SARS-CoV-2 vaccine<br>≥92 months *vs.* <92 months	0.92	0.59-1.43	0.72				1.09	0.65-1.82	0.74			
Lymphocyte count<br>≥ 5.00 x 10^9/L *vs.* <5.00 x10^9/L	1.39	0.90-2.16	0.14				1.36	0.79-2.32	0.26			
IgG levels, mg/dl<br><550 *vs.* >550	0.99	0.62-1.59	0.96				1.08	0.63-1.86	0.77			
Beta-2 microglobulin mg/dl<br>≥ 3.5 *vs.* <3.5	0.82	0.42-1.60	0.56				1.18	0.59-2.36	0.64			
Rai stage<br>III-IV *vs.* 0-II	0.90	0.33-2.47	0.84				0.98	0.24-4.02	0.97			
Clinical signs of progressive disease<br>present *vs.* absent	1.33	0.73-2.41	0.35				1.26	0.50-3.14	0.63			
IGHV<br>unmutated *vs.* mutated	0.90	0.57-1.43	0.66				0.87	0.51-1.48	0.61			
*TP*53 deletion and/ or mutation<br>absent *vs.* present	**1.79**	**1.03-3.11**	**0.038**	**1.85**	**1.06-3.25**	**0.032**	1.71	0.97-3.01	0.065	**1.90**	**1.05-3.44**	**0.035**
Prior treatment<br>yes *vs.* no	1.00	0.56-1.79	0.99									
Number of prior treatments<br>≥2 *vs.* 1	**1.55**	**1.00-2.41**	**0.050**	**2.08**	**1.27-3.40**	**0.004**	**1.67**	**0.99-2.83**	**0.055**	**2.05**	**1.19-3.53**	**0.009**
Months from last rituximab<br>≤12 *vs.* o rituximab or >12	1.63	0.96-2.76	0.070				1.63	0.93-2.83	0.086			
Ibrutinib based-therapy<br>*vs* venetoclax-based therapy							1.19	0.72-1.98	0.49			
Months from the start of Ibrutinib or venetoclax-based therapy ≥ 18 *vs.* <18							**0.38**	**0.19-0.75**	**0.005**	**0.31**	**0.15-0.63**	**0.001**

Abbreviations: Covid-19, Coronavirus ***disease*** 2019; SARS-CoV-2, severe acute respiratory syndrome *coronavirus 2;* Ig, immunoglobulins; *TP*53 gene, tumor protein p53 gene; Del., deletion; IGHV, immunoglobulin heavy chain variable region gene.
